# Circulating let-7f-5p improve risk prediction of prostate cancer in patients with benign prostatic hyperplasia

**DOI:** 10.7150/jca.45077

**Published:** 2020-05-18

**Authors:** Yuqiu Ge, Qiangdong Wang, Wei Shao, You Zhao, Qianqian Shi, Qinbo Yuan, Li Cui

**Affiliations:** 1 Department of Public Health and Preventive Medicine, Wuxi School of Medicine, Jiangnan University, Wuxi, China.; 2 Department of Urology, Huaiyin Hospital of Huai'an City, Huai'an, China.; 3 Department of Urology, Huaiyin People's Hospital of Huai'an City, Huai'an, China.; 4 Department of Science and Technology, Sir Run Run Hospital, Nanjing Medical University, Nanjing, China.; 5 Department of Urology, The Third Affiliated Hospital of Soochow University, Changzhou, China.

**Keywords:** microRNA, prostate cancer, benign prostatic hyperplasia, diagnostic biomarker

## Abstract

**Background**: Although the prostate-specific antigen (PSA) testing was widely used for early detection of prostate cancer (PCa), it is difficult for PSA to distinguish the PCa from benign prostatic hyperplasia (BPH) patients. Emerging evidence has shown that microRNA (miRNA) was a promising biomarker for PCa screening.

**Methods**: We applied miRNA profiling from microarray or high-throughput sequencing in Gene Expression Omnibus (GEO) and The Cancer Genome Atlas (TCGA) databases to identify the differentially expressed miRNAs in PCa patients (n = 1,017) and controls (n = 413). Then, qRT-PCR analysis was used to validate the expression of candidate miRNAs in our independent cohort, include 66 PCa cases and 63 BPH patients diagnosed by biopsy. The area under the receiver operating characteristic curve (AUC) was conducted to evaluate the diagnostic efficacy of miRNAs and PSA.

**Results**: In the microarray analysis, we identified two consistently differently expressed miRNAs (miR-103a-3p and let-7f-5p) between PCa patients and controls. In the subsequent qRT-PCR analysis, the let-7f-5p was upregulated in PCa compared with BPH patients (*P*=2.17E-07), but no statistically difference of miR-103a-3p expression was observed (*P*=0.456). The AUC was 0.904 for combination of lef-7f-5p and PSA, which was significantly higher than that of let-7f-5p (0.782) or PSA (0.795) alone (*P*=7.55E-04 and *P*=2.09E-03, respectively). Besides, the results of decision curve analysis and nomogram prediction indicated that combination of let-7f-5p and PSA had superior predictive accuracy of PCa.

**Conclusions**: Our study suggests that plasma let-7f-5p combining PSA could serve as potentially diagnostic biomarkers for PCa.

## Introduction

Globally, prostate cancer (PCa) is the third common cancer for men with 1.33 million cases and 0.42 million deaths in 2017^1^. It was reported that the incidence of advanced and metastatic PCa was increased in the United States from 2007^2^. In recent years, prostate-specific antigen (PSA) was widely used in early detection of PCa and had an effect on the reduction of metastatic PCa at diagnosis 3. However, PSA is still a controversial biomarker for its limited specificity and low negative predictive value for antidiastole of PCa versus benign disease 4. The new biomarkers for improvements of PCa screening were urgent.

It is well established that small non-coding RNAs known as microRNAs (miRNAs) are involved in the carcinogenesis of cancer by altering the post-transcriptional regulation 5. The miRNAs participated in a diversity of biologic processes, such as cell differentiation, proliferation and apoptosis 6, 7. The aberrant miRNAs were often observed in multiple cancers 8. There were many deregulated miRNAs in PCa tissues, for example downregulation of the miR-205 9 or miR-143/145 cluster 10, and upregulation of miR-21 11 or miR-183 family 12. For the important functions of miRNAs, mounting evidence supported that miRNAs may act as diagnostic and prognostic biomarkers for PCa 13, 14. Recently, Luo *et al.* found that miR-1246 was a promising biomarker for diagnosis and monitor the aggressiveness of PCa 15. Another study revealed that miRNAs combined with PSA testing may increase the accuracy of PCa detection 16. To identify PCa-associated miRNAs and assess its diagnostic value may provide beneficial information for screening and management of PCa patients.

In the present study, we used multiple high-sequencing miRNA profiles or miRNA array files in public databases, including Gene Expression Omnibus (GEO) and The Cancer Genome Atlas (TCGA), to systematically screen differentially expressed miRNAs (DEMIs) between PCa cases and cancer-free controls. Furthermore, we validated the expression of these significant miRNAs in independent PCa and benign prostatic hyperplasia (BPH) patients and assessed their discernibility ability of PCa detection.

## Materials and Methods

### Clinical samples and ethical statement

A total of 66 PCa and 63 BPH plasma samples were acquired from Changzhou hospital between Sep, 2017 and Aug, 2018. The diagnosis of PCa and BPH were confirmed by two clinical pathologists using biopsy. All of the subjects have written the informed consents. The Institutional Review Board of The Third Affiliated Hospital of Soochow University approved this study.

### Data availability

In the discovery phase, the miRNA microarrays in GEO dataset (GSE112264 17 and GSE113234 16) were used to identify differentially expressed circulating miRNAs between PCa and cancer-free samples. Then we applied the other independent miRNA array datasets to identify and validate the consistently significant miRNAs. GSE113486 18 and GSE60117 19 were used in the first validation phase (validation I). The miRNA expression by RNAseq from TCGA database, downloaded in UCSC Xena project (http://xena.ucsc.edu/), was used in the second validation phase (validation II). The detail information of GEO and TCGA datasets is summarized in Supplementary Table 1.

### Measurement of markers in human plasma

Quantitative real-time PCR (qRT-PCR) analysis was applied to detect the expression of candidate miRNAs in our plasma samples. The RNA was extracted from 100 μL of plasma using QIAGEN miRNeasy Mini Kit (Qiagen, CA, USA) according to the instructions in the manual. We applied Takara RNA PCR Kit (AMV) to conduct miRNA reverse transcription. The qRT-PCR was performed using SYBR(R) PrimeScript™ RT-PCR Kit (Takara Bio). The ABI 7900HT Real-Time PCR System (Applied Biosystem, Foster City, CA, USA) was used to detect the expression of candidate miRNAs and the internal reference was U6. Raw data were exported using SDS software and analyzed with the RQ manager software (Applied Biosystems, Foster City, CA, USA). The reverse transcription primer and PCR primers sequence of candidate miRNAs (miR-103a-3p and let-7f-5p) and U6 are shown in Supplementary Table 2.

### Statistical analysis

Continuous variables were described as mean ± standard deviation, and student's *t* test was conducted to assess the differences of miRNA expression between two groups. Univariate and multivariable logistic regression models were applied to assess the effects of let-7f-5p, PSA alone and combination of let-7f-5p and PSA. We calculated the area under the curve (AUC) and corresponding 95% confidence interval (95%CI) to evaluate the diagnostic efficacy of let-7f-5p and PSA. To investigate whether the AUC of let-7f-5p, PSA alone and their combination were different, DeLong's test was conducted to assess the difference in AUC between two models. Decision Curve Analysis was applied to compare the clinical net benefit of different prediction models. We used the nomogram to evaluate the risk of PCa and the calibration curve was used to assess predictive accuracy of monogram. In this study, two-sided* P* value less than 0.05 was treated as statistically significant. All statistical analyses were performed by R 3.6.1 software. Receiver operating characteristic (ROC) curve analysis was conducted by “pROC” package. Decision Curve Analysis was done using “rmda” package. Nomogram and calibration curve analysis was performed with “rms” package.

## Results

### Screening of candidate miRNAs for PCa

**Figure 1:**
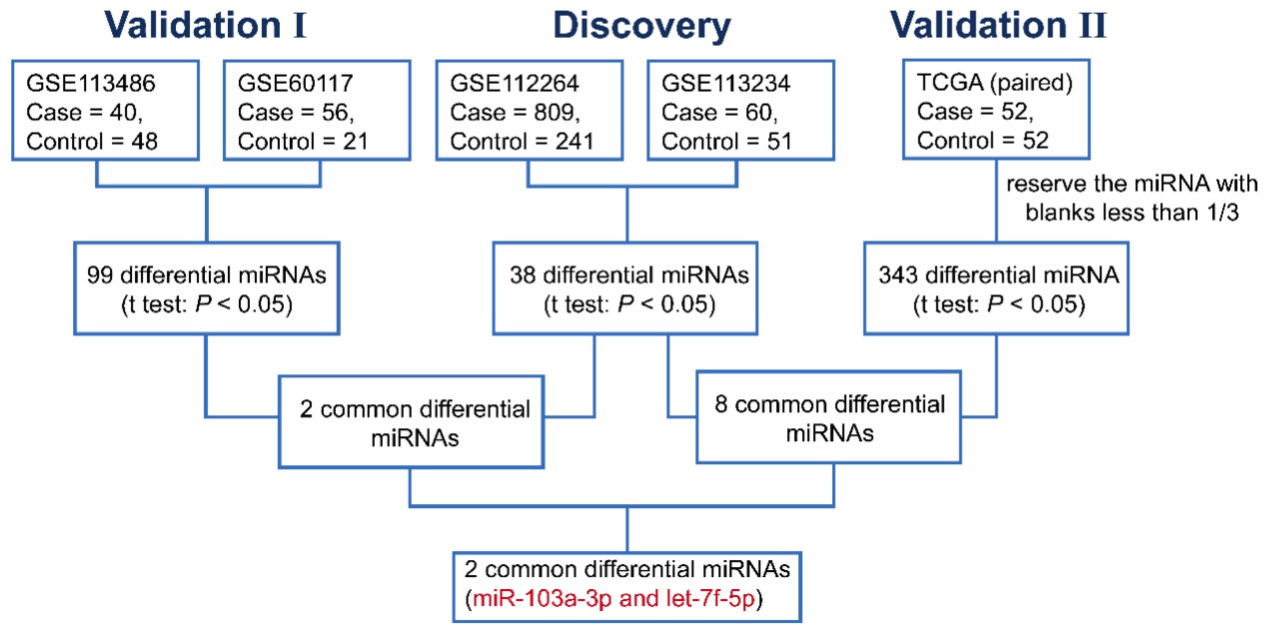
The flow chart for selecting candidate miRNAs in PCa.

**Figure 2:**
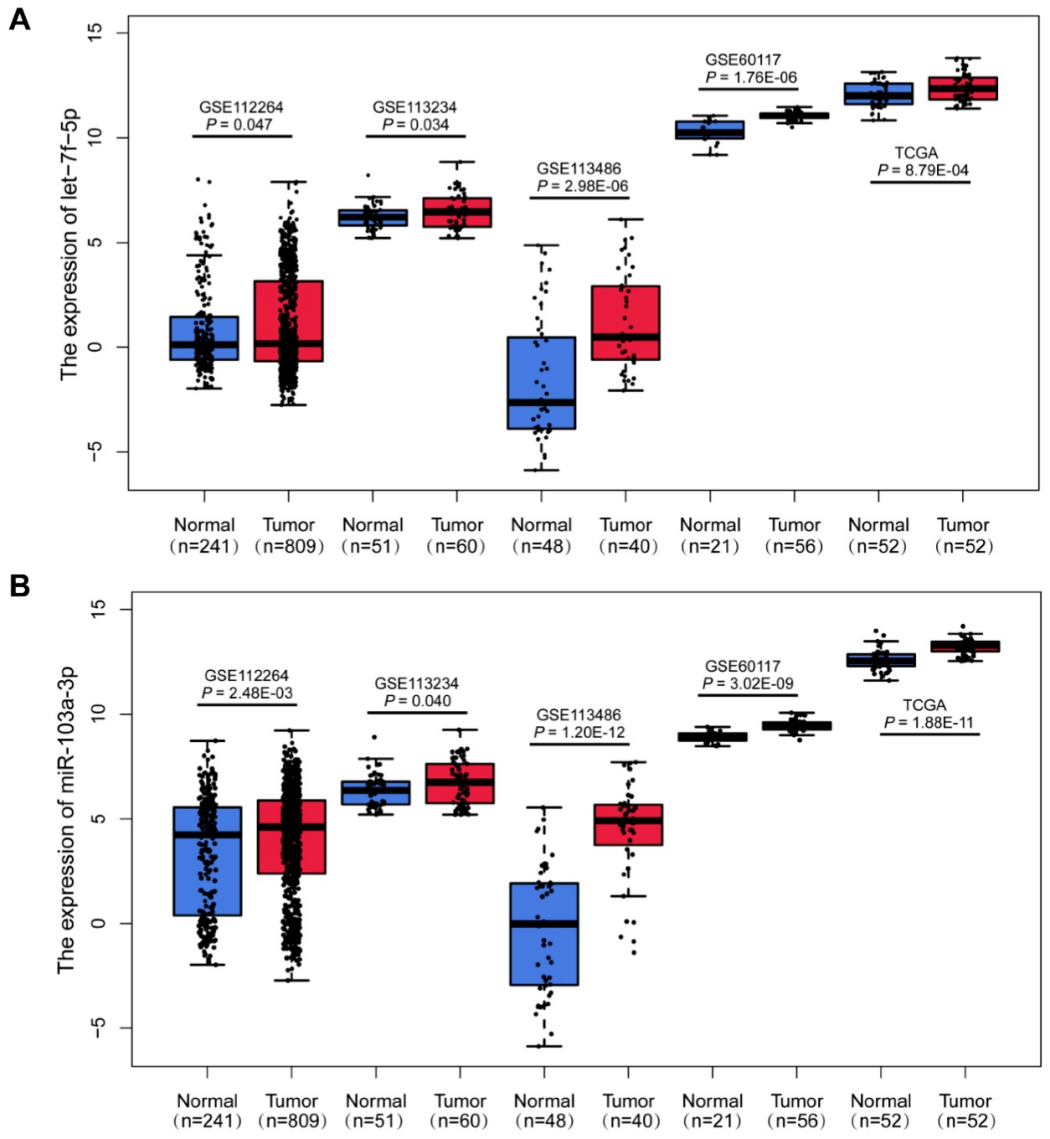
** Upregulation of miR-103a-3p and let-7f-5p in PCa.** Boxplots of let-7f-5p (A) and miR-103a-3p (B) expression in PCa patients and cancer-free controls from GSE112264, GSE113234, GSE113486, GSE60117 and TCGA datasets.

In the discovery phase, we downloaded the miRNA expression profiles from GEO database (GSE112264; GSE113234), and identified 38 differentially expressed miRNAs (DEMIs) between PCa cases and controls in the both datasets (*P*<0.05). In the following validation I, the other two GEO datasets (GSE113486; GSE60117) were analyzed and 99 DEMIs were observed. Among them, 2 miRNAs were significantly dysregulated in both discovery and validation I phase. Furthermore, miRNA expression from TCGA RNAseq dataset of 52 paired PCa tissues and adjacent normal tissues was acquire (validation II). And there were 343 DEMIs between PCa tumor and adjacent normal tissues and 8 consistently dysregulated miRNAs were found in both discovery and validation II phase. Altogether, we integrated and acquired the consistently dysregulated miRNAs between discovery, validation I and validation II phases. Two consistently differential miRNAs miR-103a-3p and let-7f-5p were screened by microarray and RNAseq analysis (Figure 1). As shown in Figure 2, the expression level of miR-103a-3p and let-7f-5p were both significantly higher in PCa patients than that in cancer-free controls (*P*<0.05).

### Validation of miR-103a-3p and let-7f-5p in our plasma samples

**Figure 3:**
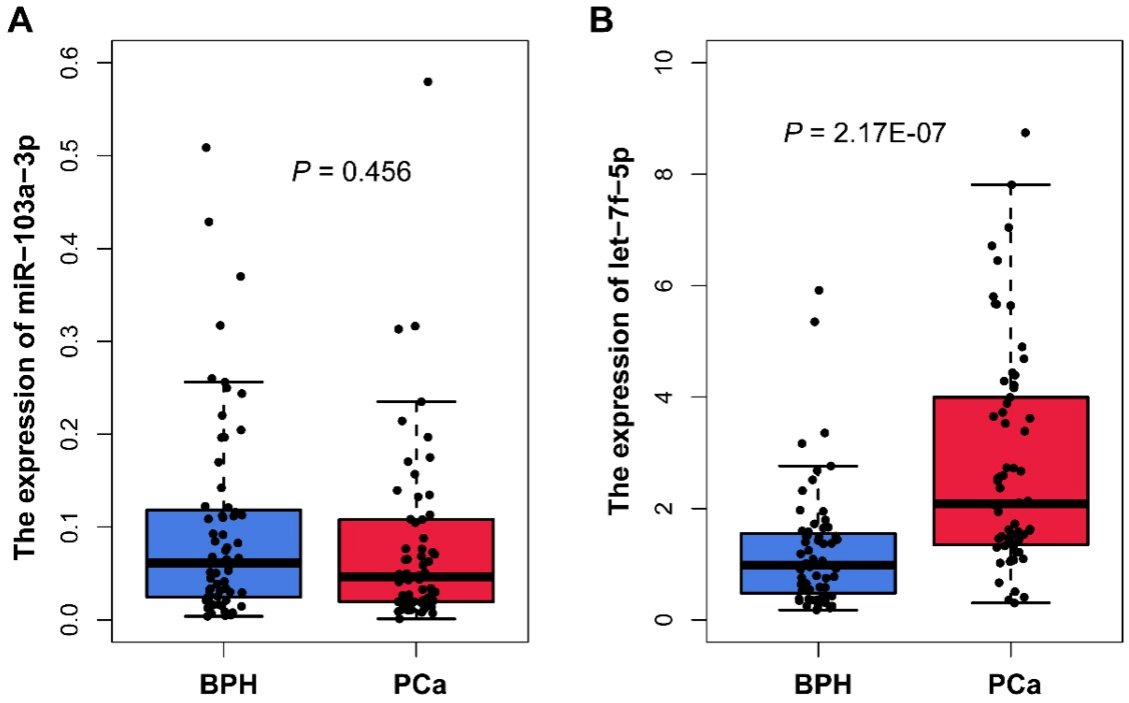
Plasma miR-103a-3p and let-7f-5p expression from patient with PCa and BPH in our cohort.

We measured the miR-103a-3p and let-7f-5p expression in plasma samples from 66 PCa and 63 BPH patients using qRT-PCR analysis. The characteristics of samples are summarized in Table 1. The results showed that there was no statistically difference in miR-103a-3p expression between PCa and BPH patients (*P*=0.456; Figure 3A). For another candidate miRNA let-7f-5p, the result of qRT-PCR was consistency with the microarray analysis. We found significant higher expression of let-7f-5p in PCa plasma, as compared with BPH patients (*P*=2.17E-07; Figure 3B). We also evaluated whether the let-7f-5p was related to the clinical features of PCa patients recruited in this study. The results indicated that the expression level of let-7f-5p was slightly associated with the age of patient (*P*=0.064), and no significant associations were observed in stage, gleason score, PSA level and bone metastasis status (Table 2).

### Diagnostic value of let-7f-5p and PSA for PCa

**Figure 4:**
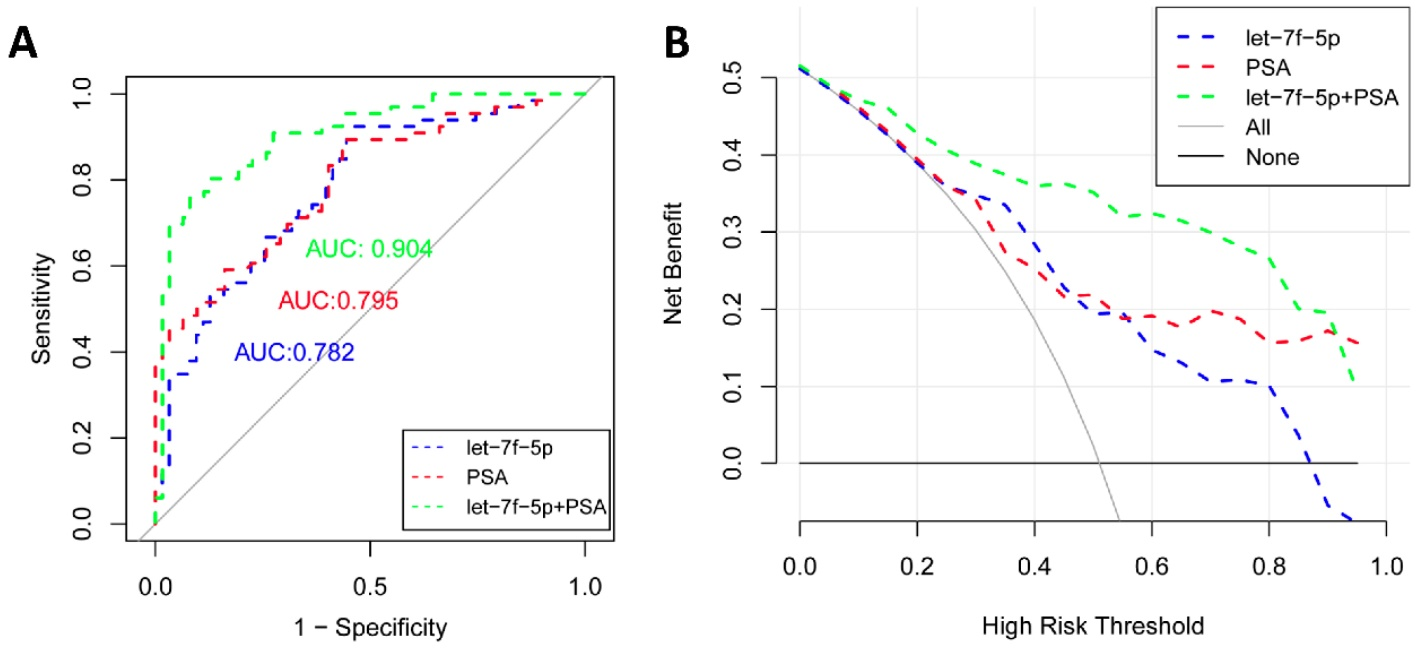
** The ROC curve and decision curve analysis for prediction models of PCa and BPH patients.** (A) ROC curves for let-7f-5p, PSA, and combination of let-7f-5p and PSA expression in plasma samples from PCa and BPH patients. (B) Decision curve analysis was applied to compare the net benefit between different prediction models. The y-axis means the net benefit. The blue dashed line: model including let-7f-5p only; The red dashed line: model including PSA only; The green dashed line: model including let-7f-5p and PSA; The grey solid line means all subjects at risk; The black solid line means none subjects at risk.

**Figure 5:**
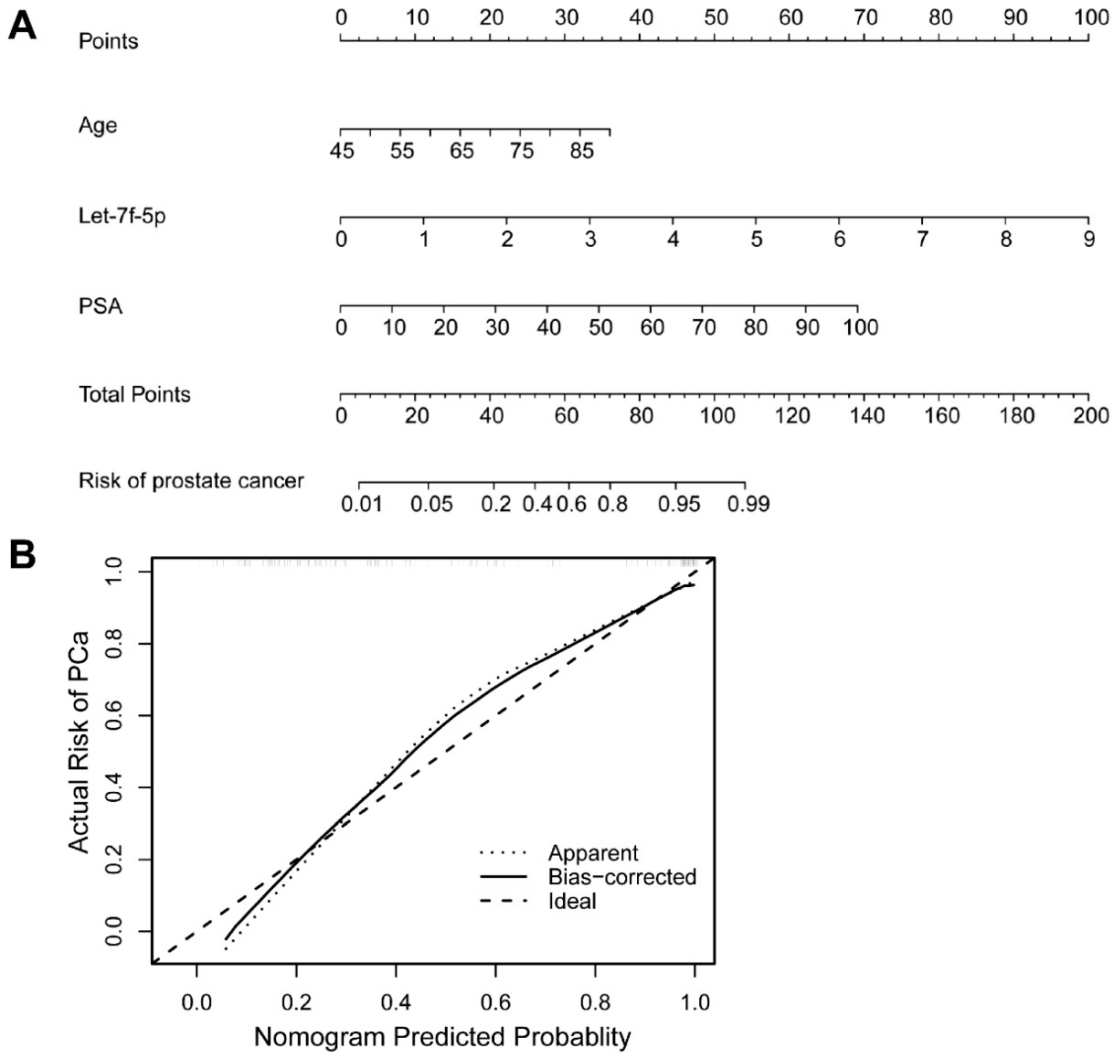
** A nomogram and calibration curve for predicting PCa risk integrated with age, let-7f-5p and PSA levels.** (A) Nomogram of logistic regression model for PCa. The nomogram enabled the user to obtain the probability of PCa risk corresponding to a patient's combination of covariates. The upper-most point scale represents the predicator points of each variable. Then the user can add up the points and read the corresponding predicted values at the bottom of the nomogram. (B) Calibration curve of the nomogram for predicting PCa risk. The calibration curve represents the agreement between the nomogram predicted risk probability (x-axis) and the actual risk probability of PCa (y-axis). The diagonal dotted line is the ideal curve. The other dotted line means the predictive performance of nomogram. The black line represents bias-corrected curve.

As shown in Figure 4A, the ROC curve analyses were applied to assess the ability of significantly expressed miRNA let-7f-5p and PSA to discriminate the PCa and BPH patients. The AUC and 95%CI for all models are summarized in Table 3. For let-7f-5p alone, the AUC is 0.782 (95%CI: 0.703-0.861). For PSA alone, the AUC is 0.795 (95%CI: 0.720-0.871). Importantly, for the combination of let-7f-5p and PSA, the AUC is 0.904 (95%CI: 0.852-0.957). The DeLong's test was conducted to evaluate whether the AUC were different between two models. The results indicated that the AUC of combination of lef-7f-5p and PSA was significantly higher than that of let-7f-5p or PSA alone (*P*=7.55E-04 and *P*=2.09E-03, respectively). The decision curve analysis also showed that combination of let-7f-5p and PSA model had best performance in risk predication and clinical benefit among the three models (Figure 4B). In addition, we applied a nomogram to predict the PCa risk using the variables of age, let-7f-5p and PSA levels and the concordance index reached up to 0.921(Figure 5A). The calibration plot demonstrated the agreements between the predicted probability of PCa using nomogram and observed risk of PCa. Our results indicated good consistency between predictive and actual PCa risk, suggesting the high predictive accuracy of the nomogram for PCa risk (Figure 5B).

## Discussion

PSA testing decreased the mortality of PCa but was related to a high risk of overdiagnosis^20^. It is difficult for PSA to distinguish the PCa from BPH patients, which may contribute to a mass of misdiagnose and unnecessary biopsies 21. Therefore, improved biomarkers for detection of PCa remain necessary 22.

The circulating miRNAs were stable and could be treated as non-invasive biomarkers 23. Compelling evidence indicates that miRNA can act as potential diagnostic biomarker in cancers, including PCa 24. Recently, microarray or high-throughput sequencing has been extensively applied to measure the expression of miRNAs and identify the differential expressed miRNAs. In the present study, through integration of multiple miRNA expression profiles from GEO and TCGA databases, we identified two miRNAs, miR-103a-3p and let-7f-5p, consistently upregulated in PCa patients. It is noteworthy that we used both miRNA profiling by microarray or high-throughput sequencing technology, and the datasets include miRNA expression in plasma and tissue of PCa cases and cancer-free controls. Regardless of the sample sizes and the platforms of datasets varied, these two miRNAs (miR-103a-3p and let-7f-5p) were significantly dysregulated in PCa cases from all datasets. Furthermore, a significant higher expression of let-7f-5p was also observed in PCa cases, compared with BPH patients in our cohort. The expression of candidate miRNA miR-103a-3p had no remarkable difference between groups. Another study was in inconsistence with our results. It was reported that miR-103a-3p was upregulated in PCa and had good detectability of overall and clinically significant PCa 16. This controversial result should be investigated in the further study.

The result of the microarray and qRT-PCR analysis confirmed that expression level of let-7f-5p was upregulated in PCa. To our knowledge, the association between miRNA let-7f-5p and PCa has not been reported. The role of let-7f-5p have investigated in other diseases. The expression level of let-7f-5p was upregulated in colon cancer 25, 26, thyroid cancer cells 27, typical and atypical carcinoid tumors 28. However, downregulation of let-7f-5p was observed in amyotrophic lateral sclerosis 29. Besides, a recent study found that let-7f-5p promoted the resistance of colorectal cancer cells and silence of let-7f-5p may provide a novel therapeutic strategy for colorectal cancer 26. The role of let-7f-5p acted as biomarker was also explored. A previous study revealed that let-7f-5p was a prognostic biomarker for chemoresistance in adenocarcinomas of the esophagogastric junction 30. In addition, Marcia *et al* found that let-7f-5p combined with other four miRNAs showed high diagnostic accuracy for Parkinson's disease 31.

The limitations of PSA have promoted investigation of new biomarkers for detection of PCa, which can reduce unnecessary biopsies 32. Mounting evidence has shown that combination of new biomarker and PSA may improve the predictive accuracy of PSA testing in diagnosis of PCa 33. In the present study, we determined the effect of let-7f-5p and PSA on diagnose of PCa. The results showed that the AUC of PSA was slightly higher than that of let-7f-5p. Moreover, combined panel of let-7f-5p and PSA could discriminate PCa patients from BPH subjects with higher performance than PSA alone, suggesting this panel can improve the diagnostic route of PSA testing.

The present study has several important strengths. Firstly, we acquired and downloaded multiple large-scale miRNA datasets from a variety of platforms (1,017 cases, 413 controls). Through integrative analysis of database of GEO and TCGA, we systematically screened and identified two candidate miRNAs involved in PCa. Moreover, the results in microarray analysis were validated in an independent PCa cases and BPH subjects, who were confirmed by biopsy. There are some limitations in our study. The sample size in qRT-PCR analysis was relatively small, which may influence the power of detecting differentially expressed miRNAs. Besides, more multi-center case-control studies with large sample may need for further validation of the diagnostic effect of let-7f-5p and PSA.

In summary, we identified and validated that miRNA let-7f-5p was significantly upregulated in PCa. The combination of let-7f-5p and PSA had a better discernibility of PCa and BPH patients. Our study suggests that let-7f-5p and PSA panel may serve as biomarker for PCa detection. Further studies in large-scale independent population are warrant to validate and define the diagnostic effect of this panel.

## Supplementary Material

Supplementary tables.

## Figures and Tables

**Table 1:** Characteristics of PCa and BPH patients

Variables	Prostate cancer (n=66)	Benign Prostatic Hyperplasia (n=63)
Age	72.0 ±7.1	69.2 ±7.6
PSA (ng/ml)		
≤10	11 (16.7%)	36 (58.1%)
>10	55 (83.3%)	26 (41.9%)
Tumor		
T1/T2	62 (93.9%)	
T3/T4	4 (6.1%)	
Node		
N0	63 (95.5%)	
N1-3	3 (4.5%)	
Metastasis		
M0	52 (78.8%)	
M1	14 (21.2%)	
Stage		
I/II	47 (71.2%)	
III/IV	19 (28.8%)	
Gleason score		
≤7	43 (65.2%)	
>7	23 (34.8%)	
Bone metastasis		
No	44 (71.0%)	
Yes	18 (29.0%)	

**Table 2:** The distribution of let-7f-5p expression in PCa patients

Variables	Cases	let-7f-5p expression	*t* value	*P*
Age				
≤70	30	3.27±2.33	1.89	0.064
>70	36	2.38±1.50		
Stage				
I/II	47	2.82±1.95	0.22	0.828
III/IV	19	2.70±2.02		
Gleason score				
≤7	43	3.03±2.01	1.42	0.161
>7	23	2.32±1.81		
PSA (ng/ml)				
≤10	11	2.56±1.23	-0.42	0.679
>10	55	2.83±2.08		
Bone metastasis				
No	44	2.97±2.08	1.20	0.234
Yes	18	2.30±1.77		

**Table 3:** ROC analysis of let-7f-5p and PSA from patients with PCa and BPH

Factors	AUC	95%CI	*P* value
let-7f-5p	0.782	0.703-0.861	
PSA	0.795	0.720-0.871	
let-7f-5p+PSA	0.904	0.852-0.957	
let-7f-5p *vs.* let-7f-5p+PSA			7.55E-04
PSA *vs.* let-7f-5p+PSA			2.09E-03
