# Supplementary material for: Circulating let-7f-5p improve risk prediction of prostate cancer in patients with benign prostatic hyperplasia
Source: J Cancer. 2020 May 18;11(15):4542–9. doi: 10.7150/jca.45077 (PMC7255360; doi:10.7150/jca.45077)
Supplement: Supplementary file 1 — Supplementary tables. [file jcav11p4542s1.pdf]

Supplementary Table 1. The basic information of public datasets from GEO and TCGA

Datasets	Platforms	Samples
GSE112264	GPL21263: 3D-Gene Human miRNA V21_1.0.0	809 PCa cases and 241 cancer-free controls
GSE113234	GPL19730: Agilent-046064 Unrestricted_Human_miRNA_V19.0_Microarray	60 PCa cases and 51 cancer-free controls
GSE113486	GPL21263: 3D-Gene Human miRNA V21_1.0.0	40 PCa cases and 48 cancer-free controls
GSE60117	GPL3264: Agilent-021827 Human miRNA Microarray (V3)	56 PCa cases and 21 cancer-free controls
TCGA	IlluminaHiSeq_RNASeqV2	52 paired PCa cases and adjacent normal control

Supplementary Table 2. The primers of candidate miRNAs and U6

Genes	Reverse transcription primer	PCR Primers
U6	AACGCTTCACGAATTTGCGT	F: CTCGCTTCGGCAGCACA<br>R: AACGCTTCACGAATTTGCGT
miR-103a-3p	CTCAACTGGTGTCGTGGAGT<br>CGGCAATTCAGTTGATTCATAGCC	F: ACACTCCAGCTGGGAGCAGCATTGTACAGGG<br>R: TGGTGTCGTGGAGTCG
let-7f-5p	CTCAACTGGTGTCGTGGAGT<br>CGGCAATTCAGTTGATAACTATAC	F: ACACTCCAGCTGGGTGAGGTAGTAGATTGT<br>R: TGGTGTCGTGGAGTCG
